# Reference Ranges and Association of Age and Lifestyle Characteristics with Testosterone, Sex Hormone Binding Globulin, and Luteinizing Hormone among 1166 Western Chinese Men

**DOI:** 10.1371/journal.pone.0164116

**Published:** 2016-10-06

**Authors:** Xubo Shen, Ruifeng Wang, Na Yu, Yongjun Shi, Honggang Li, Chengliang Xiong, Yan Li, Ellen M. Wells, Yuanzhong Zhou

**Affiliations:** 1 School of Public Health, Zunyi Medical University, Zunyi, China; 2 Huichuan District Center for Disease Control and Prevention, Zunyi, China; 3 Institute of Family Planning, Tongji Medical College, Huazhong University of Science and Technology, Wuhan, China; 4 School of Health Sciences, Purdue University, West Lafayette, Indiana, United States of America; University of Colorado Denver School of Medicine, UNITED STATES

## Abstract

Decreased total testosterone (TT) is the recommended metric to identify age-related hypogonadism. However, average TT and the extent to which it varies by age, can vary substantially among different populations. Population-specific reference ranges are needed to understand normal versus abnormal TT levels. Therefore, the goal for this study was to describe androgen concentrations and their correlates among Western Chinese men. We completed a population-based, cross-sectional study including 227 young adults (YA) (20–39 years) and 939 older adults (OA) (40–89 years). We measured TT, sex-hormone binding globulin (SHBG), luteinizing hormone (LH), testosterone secreting index (TSI), and calculated free testosterone (cFT). Reference ranges for this population were determined using average YA concentrations. Multivariable regression models were used to predict hormone concentrations adjusting for age, waist-to-height ratio (WHR), marital status, education, occupation, smoking, alcohol, blood glucose, and blood pressure. Among OA, 3.8% had low TT, 15.2% had low cFT, 26.3% had low TSI, 21.6% had high SHBG, and 6.1% had high LH. Average cFT was significantly lower in OA (0.30 nmol/L; standard deviation (SD): 0.09) versus YA (0.37; SD: 0.11) but TT was not different in OA (16.82 nmol/L; SD: 4.80) versus YA (16.88; SD: 5.29). In adjusted models increasing age was significantly associated with increased SHBG or LH, and decreased cFT or TSI; however, TT was not significantly associated with age (β = 0.02 nmol/L; 95% confidence interval (CI): -0.01, 0.04). Higher WHR was associated with significantly decreased TT, SHBG, TSI, and LH. The only variable significantly related to cFT was age (β = -0.0033; 95% CI:-0.0037, -0.0028); suggesting that cFT measurements would not be confounded by other lifestyle factors. In conclusion, cFT, but not TT, varies with age in this population, suggesting cFT may be a better potential marker for age-related androgen deficiency than TT among Western Chinese men.

## Introduction

Late onset hypogonadism (LOH) [1] is a clinical and biochemical syndrome associated with advancing age and is characterized by sexual, psychological, and physical symptoms as well as serum testosterone concentrations below the reference range defined by typical values among young, healthy adult males [2–4]. LOH may significantly reduce quality of life and adversely affects the function of multiple organ systems [3, 5]; severe LOH is even associated with substantially higher risk of all-cause and cardiovascular mortality [6].

Descriptive epidemiology studies suggest this affects a substantial number of older men. Buvat *et al*. estimated androgen deficiency prevalence among aging men to be between 7% and 49% [7]. Incidence of total testosterone (TT) <325 ng/dL in United States males was ~20% among 60–69 year old men, ~30% among 70–79 year old men, and ~50% among men ≥ 80 years old [8]. Notably, greater incidence was found with reduced free testosterone index instead of TT [8]. Reported rates are lower when using a combination of androgen concentrations and reported symptoms, but are still of concern. A report from the Massachusetts Male Aging Study used a combination of multiple symptoms, TT, and free testosterone (FT) as diagnostic criteria reported prevalence of androgen deficiency at 5.9% among 40–49 year olds, 11.2% among 50–59 year olds, and 23.3% among 60–69 year olds [9]. Lower prevalence was reported from the European Male Aging Study: 2.1% among men between 40 and 79 years old using both androgen and symptom criteria [10].

As seen from the statistics above, age is strongly associated with androgen deficiency or LOH. In addition, lifestyle factors including obesity and smoking may also influence serum androgen concentrations [11–15]. Several studies have observed that obese men tend to have lower TT and sex hormone binding globulin (SHBG), but not FT [11–15]; tobacco consumption was positively associated with serum levels of TT and FT [11, 15].

A variety of criteria have been used in the literature to define androgen deficiency or LOH; this reflects the challenge within this field to develop detailed unified criteria. Guidelines from several professional societies (International Society of Andrology, International Society for the Study of the Aging Male, European Association of Urology, European Academy of Andrology, and American Society of Andrology) recommend decreased total testosterone as the primary marker of LOH [16, 17]. However, it is still challenging to implement this recommendation on a local level because average TT varies substantially in different populations; thus a specific concentration that might qualify as a decreased concentration within one population may not represent a decrease in another [8, 11, 14, 15, 18–22]. Additionally, several researchers have noted that free testosterone may also be of value in identifying androgen deficiency of LOH because age-related decline in free testosterone is more consistently observed and of a greater magnitude than an age-related decline in total testosterone [15, 19, 21, 22].

Although reference ranges for androgen hormones have been described for the United States [5, 23, 24], Europe [25–28], and some parts of Asia [4, 29], this information has rarely been described and still remains unclear for Chinese men [14, 30]. This information is important to help local clinicians accurately identify men who may be affected by LOH. Therefore, the goal of this research was to conduct a population-based study of Western Chinese men in order to establish average androgen concentrations as well as their correlation with age and lifestyle characteristics. This may suggest which hormones may be of most relevance for describing LOH within this population.

## Methods

We conducted a population-based, cross-sectional study among adult males from Zunyi, Guizhou Province, China in 2013. Participants completed a questionnaire and brief clinical exam; hormone concentrations were determined using a fasting blood sample. This study was approved by the Ethical Committee Review Board of Tongji Medical College in the Huazhong University of Science and Technology. All subjects provided written informed consent prior to participating in the study.

### Study Setting and Subjects

Zunyi has a population of 1.2 million people living in 80 communities located <50 kilometers from the city center. A stratified cluster design was used to select communities sampled for this study. Communities were stratified by urban, suburban, and rural status. About 10% of communities within the three strata were selected for inclusion in this study: 2 urban communities (Jiaotong, Yanhong), 2 suburban communities (Heping, Jianguo), and 3 rural communities (Gema, Tiangou, Wuxing). Physicians in each of these communities contacted adult males to inform them about the study and invite them to participate. All data for this study was collected between August 20 and September 20, 2013.

A total of 1213 Chinese adult men were identified as potential study participants. Study exclusion criteria were based on reports from previous studies [16]. Additionally, criteria for this study are identical to other, ongoing studies in China [31]; this intentional design will improve our ability to compare results across the entire country in the future. Inclusion criteria were male sex, age between 20–89 years, and residence in one of the communities selected for inclusion in this study. Men were excluded for the following reasons: diagnosed cardiac, kidney, or liver disease (n = 11), pituitary-testicular disease (n = 1), drug abuse (n = 4). Additional exclusion criteria include incomplete or illogical questionnaire responses (n = 29) and insufficient or non-fasting blood sample (n = 2). After exclusions, 1166 subjects (1166/1213, 96.12%) participated in this study.

### Questionnaire and Physical Examination

All questionnaire, physical exam, and blood sample data were anonymized using a participant identification code to help protect the confidentiality of study participants.

Participants completed a questionnaire designed to collect information on demographics and socioeconomic status. We assessed age, medical history, smoking, alcohol use, and obesity as previous studies have identified these as potentially significant factors in hormone concentrations [11, 13, 26, 32]. Occupation, marital status, and years of education were additional variables used in this study to classify socioeconomic characteristics. Variables were categorized using standard clinical classifications or sociodemographic attributes. A male physician was present to assist with the questionnaire completion upon participant request; no other individuals were present for questionnaire completion.

A physical examination was conducted by trained study staff to measure height, weight, waist circumference, systolic blood pressure (SBP), and diastolic blood pressure (DBP). Body mass index (BMI) (a measure of general obesity) and waist-to-height ratio (WHR) (a measure of central obesity) were calculated as: BMI = weight (kg) / (height (m))^2^ and WHR = waist circumference (cm) / height (cm). A cutoff value of 0.5 (≤ 0.5 = normal weight; >0.5 = overweight or obese) was used for WHR, as recommended previously [33].

Trained nurses collected blood samples from 7:00 to 11:00 AM following an overnight fast. Although testosterone concentrations do vary diurnally, this time period represents peak or near-peak concentrations [34], and this time period is therefore recommended by the International Society of Andrology, the International Society for the Study of the Aging Male, the European Association of Urology, European Association of Andrology, and the American Society of Andrology [16] to collect blood for testosterone evaluation. Venous blood was centrifuged for 15 min at 4°C to obtain serum within 1 hour, and serum was stored at -80°C until analysis. Whole blood was used to assess blood glucose using standard clinical methods.

### Serum Hormone Assessments

TT, SHBG and luteinizing hormone (LH) were measured by chemiluminescent immunoassays on a Beckman Access Immunoassay system (Beckman Coulter, Inc., Brea, CA, USA). It is difficult and expensive to directly measure FT; however, calculated free testosterone (cFT) is highly correlated with FT and has been used in previous research studies [35, 36]. cFT was quantified using Vermeulen’s formula [35]. Testosterone secreting index (TSI) was defined as TT (nmol/L) / LH (IU/L).

### Statistical Analyses

Questionnaire and hormone data were proofread and entered in EPIdata version 3.02 (Odense, Denmark), analyses were performed with SPSS version 18.0 (Armonk, NY, USA), and graphics were produced using Stata 13.0 (College Station, TX, USA). A *p*-value <0.05 was considered statistically significant.

Methods to determine average reference hormone concentrations were based on published examples or recommendations from the Japanese Urological Association [37]. Participants were classified as younger adults (YA) (20–39 years) or older adults (OA) (40–89 years). The group of YA was used to estimate reference concentrations for hormones within this population. The reference range was calculated using a nonparametric method: abnormal hormone concentrations were defined as values below the 5^th^ percentile for TT, cFT and TSI [16] and concentrations higher than the 95^th^ percentile for SHBG [13] and LH [38]. Reference concentrations, abnormal cutoffs, and the percentage of OA considered to be abnormal are presented.

The influence of age and other sociodemographic variables on hormone concentrations was assessed using linear regression models. Bivariate associations between hormone concentrations and demographic or lifestyle characteristics were assessed using unadjusted linear regression models and Spearman correlation coefficients. We created nonparametric lowess smoothing curves of each hormone versus age and stratified by high/low WHR. Stratification by weight was conducted because weight has been previously identified as an important variable influencing age-related hormone change [11]. We used Spearman correlation coefficients to assess the potential for multicollinearity between age and lifestyle variables. A high correlation (Spearman’s ρ>0.7, *p*<0.01) was found for BMI with WHR and SBP with DBP; therefore, we excluded BMI and DBP from multivariable analysis as WHR and SBP are thought to be better predictors of future health status [39, 40].

Multivariable linear models were created for each hormone as a dependent variable, and our *a priori* list of demographic and lifestyle characteristics as independent variables. Independent variables for each model included age (continuous), WHR (≤0.5 vs. >0.5), blood glucose (continuous), systolic blood pressure (SBP) (continuous), occupation (farmer vs. not), education level (≤5 vs. >5 years), marital status (married vs. not), smoking status (current vs. never or former), alcohol use (current vs. never or former), and history of vasectomy (yes vs. no).

## Results

### Demographic and Lifestyle Characteristics

This study included 227 YA and 939 OA (Table 1). Overall, participants’ average age was 51.6 years, 82.0% were farmers, 93.5% were married, 78.8% were current smokers, and 57.3% currently drink alcohol. OA had significantly higher average WHR (0.51; standard deviation (SD) = 0.08), SBP (130.64 mmHg, SD = 19.54), and DBP (84.43 mmHg, SD = 12.28) compared to YA, where average WHR = 0.50 (SD = 0.06), SBP = 121.27 mmHg (SD = 14.58), and DBP = 81.16 mmHg (SD = 10.56); *p*<0.05 for all comparisons. The proportion of men who were farmers, had less education, current smokers, had a vasectomy, or never/former drinkers was significantly higher among OA compared to YA; *p*<0.05 for all comparisons. There was no statistically significant difference for the remaining characteristics between OA and YA.

**Table 1:** Selected characteristics among young adults (n = 227), older adults (n = 939) and the total study population (n = 1166).

Characteristic, statistic	Category	Young Adults (20–39 years)	Older Adults (40–89 years)	Total Cohort (20–89 years)
Age in years, mean (SD)		34.93 (3.45)	55.58 (10.87)	51.56 (12.82)
BMI in kg/m^2^, mean (SD)		24.08 (3.44)	23.98 (6.22)	24.00 (5.78)
WHR, mean (SD)*		0.50 (0.06)	0.51 (0.08)	0.51 (0.08)
Glucose in mmol/L, mean (SD)		5.28 (0.84)	5.61 (1.65)	5.56 (1.56)
SBP in mmHg, mean (SD)*		121.27 (14.58)	130.64 (19.54)	128.82 (19.04)
DBP in mmHg, mean (SD)*		81.16 (10.56)	84.43 (12.28)	83.79 (12.03)
Occupation, N (%)*				
	Farmer	156 (68.7)	800 (85.2)	956 (82.0)
	Other occupation	71 (31.3)	139 (14.8)	210 (18.0)
Education, N (%)*				
	≤ 5 years	5 (2.2)	106 (11.3)	111 (9.5)
	> 5 years	222 (97.8)	833 (88.7)	1055 (90.5)
Marital status, N (%)				
	Married	208 (91.6)	882 (93.9)	1090 (93.5)
	Single, divorced or widowed	19 (8.4)	57 (6.1)	76 (6.5)
Smoking status, N (%)*				
	Current	162 (71.4)	757 (80.6)	919 (78.8)
	Never or former	65 (28.6)	182 (19.4)	247 (21.2)
Alcohol use, N (%)*				
	Current	155 (68.7)	512 (54.5)	668 (57.3)
	Never or former	71 (31.3)	427 (45.5)	498 (42.7)
Vasectomy, N (%)*				
	Yes	9 (3.9)	78 (8.3)	87 (7.5)
	No	218 (96.1)	861 (91.7)	1069 (92.5)

SD = standard deviation; BMI = body mass index; WHR = waist-to-height ratio; SBP = systolic blood pressure; DBP = diastolic blood pressure

* *p* < 0.05, likelihood ratio test, comparing young to older adults

### Hormone Concentrations and Reference Values

Reference concentrations for hormones, based on YA averages, are presented in Table 2. Among YA, average hormone concentrations were TT: 16.9 nmol/L (SD: 5.3); SHBG: 31.7 (SD: 14.6); cFT: 0.4 nmol/L (SD: 0.11); TSI: 4.8 (SD: 2.4) and LH: 4.2 IU/L (SD: 2.3). The lower cutoff values for TT, cFT and TSI were 9.16 nmol/L, 0.22 nmol/L and 1.89nmol/L, respectively; whereas the higher cutoff values for SHBG and LH were 58.26 nmol/L and 15.35 IU/L, respectively.

**Table 2:** Mean, median and interquartile range of hormone concentrations among young adults (n = 227) and calculated cutoff values.

Hormone	Mean (SD)	Median	Interquartile range	Cutoff value
TT, nmol/L	16.88 (5.29)	16.34	13.61, 19.28	9.16*
SHBG, nmol/L	31.67 (14.59)	30.10	21.60, 38.10	58.26#
cFT, nmol/L	0.37 (0.11)	0.36	0.30, 0.41	0.22*
TSI, TT/LH	4.76 (2.39)	4.35	3.25, 5.84	1.89*
LH, IU/L	4.20 (2.29)	3.73	2.82, 4.93	15.35#

Young adults = 20–39 year old; SD = standard deviation; TT = total testosterone; SHBG = sex hormone binding globulin; cFT = calculated free TT = total testosterone; TSI = testosterone secreting index; LH = luteinizing hormone. Interquartile range: 25^th^ percentile; 75^th^ percentile.

*5^th^percentile;

^#^95^th^ percentile.

Average hormone concentrations for the entire study population are presented in Table 3. Average serum TT concentration were 16.83 nmol/L (SD = 4.90) overall; 16.88 nmol/L (SD = 5.29) among YA; and 16.82 nmol/L (SD = 4.80) among OA. There was no difference in TT among YA versus OA. Meanwhile, cFT was significantly lower among OA versus YA: this was 0.37 nmol/L (SD = 0.11) among YA and 0.30 nmol/L (SD = 0.09) among OA. TSI were also significantly lower among OA versus YA whereas LH and SHBG were significantly increased among OA versus YA.

**Table 3:** Mean (SD) hormone concentrations, stratified by selected characteristics, n = 1166.

Characteristic	Category	N	TT, nmol/L	SHBG, nmol/L	cFT, nmol/L	TSI, no units	LH, IU/L
Full cohort		1166	16.83 (4.90)	42.15 (20.21)	0.31 (0.10)	3.40 (2.04)	6.77(5.14)
Age, years	< 40	227	16.88 (5.29)	31.67 (14.59)*	0.37 (0.11)*	4.76 (2.39)*	4.20 (2.29)*
	≥ 40	939	16.82 (4.80)	44.67 (20.60)*	0.30 (0.09)*	3.07 (1.79)*	7.40 (5.43)*
WHR, no units	≤ 0.5	563	18.48 (4.92)*	48.52 (20.49)*	0.32 (0.10)	3.65 (2.22)*	7.18 (5.53)*
	> 0.5	603	15.30 (4.36)*	36.20 (18.04)*	0.31 (0.10)	3.17 (1.82)*	6.39 (4.72)*
BG, mmol/L	≤ 6.2	961	17.07 (4.80)*	42.03 (19.23)	0.32 (0.10)*	3.50 (2.09)*	6.64 (5.01)
	> 6.2	205	15.69 (5.21)*	42.68 (24.33)	0.29 (0.10)*	2.96 (1.71)*	7.41 (5.68)
SBP, mmHg	≤ 130	714	17.35 (4.94)*	42.41 (20.11)	0.32 (0.10)*	3.67 (2.13)*	6.29 (4.48)*
	> 130	452	16.02 (4.73)*	41.74 (20.39)	0.30 (0.09)*	2.97 (1.80)*	7.54 (5.95)*
Occupation	Farmer	956	17.00 (4.91)*	43.58 (20.60)*	0.31 (0.09)*	3.34 (2.01)*	7.03 (5.41)*
	Other	210	16.08 (4.78)*	35.63 (16.92)*	0.33 (0.11)*	3.70 (2.13)*	5.63 (3.41)*
Education, years	≤ 5	111	17.48 (4.64)	52.55 (22.90)*	0.29 (0.12)*	2.42 (1.29)*	9.96 (7.60)*
	> 5	1055	16.76 (4.92)	41.05 (19.60)*	0.32 (0.09)*	3.51 (2.07)*	6.44 (4.69)*
Marital status	Married	1090	16.75 (4.86)*	41.90 (20.22)	0.31 (0.10)	3.41 (2.03)	6.64 (4.84)*
	Not married	76	17.95 (5.40) *	45.65 (19.95)	0.32 (0.10)	3.27 (2.08)	8.70 (8.13)*
Smoking	Never or former	247	16.31 (5.04)	40.02 (21.78) *	0.32 (0.11)	3.38 (1.90)	6.95 (5.98)
	Current	919	16.97 (4.85)	42.72 (19.74)*	0.31 (0.10)	3.41 (2.07)	6.73 (4.89)
Alcohol use	Never or former	498	17.00(4.70)	44.78 (20.94)*	0.31 (0.09)	3.16(1.80)	7.42 (5.89)*
	Current	668	16.70(5.04)	40.18 (19.44)*	0.32 (0.10)	3.59 (2.18)	6.29 (4.43)*
Vasectomy	Yes	87	16.71 (4.93)	44.65 (21.53)	0.31 (0.12)	2.99 (1.62)	7.60 (6.28)
	No	1079	16.83 (4.91)	41.99 (20.17)	0.31 (0.10)	3.43 (2.07)	6.71 (5.02)

TT = total testosterone; SHBG = sex hormone-binding globulin; cFT = calculated free testosterone; TSI = testosterone secreting index; LH = luteinizing hormone; WHR = waist-to-height ratio; BG = Blood glucose; SBP = systolic blood pressure; SD = standard deviation; Not married = single or divorced or widowed.

*p < 0.05, likelihood ratio test, comparing young to older adults

**Fig 1:**
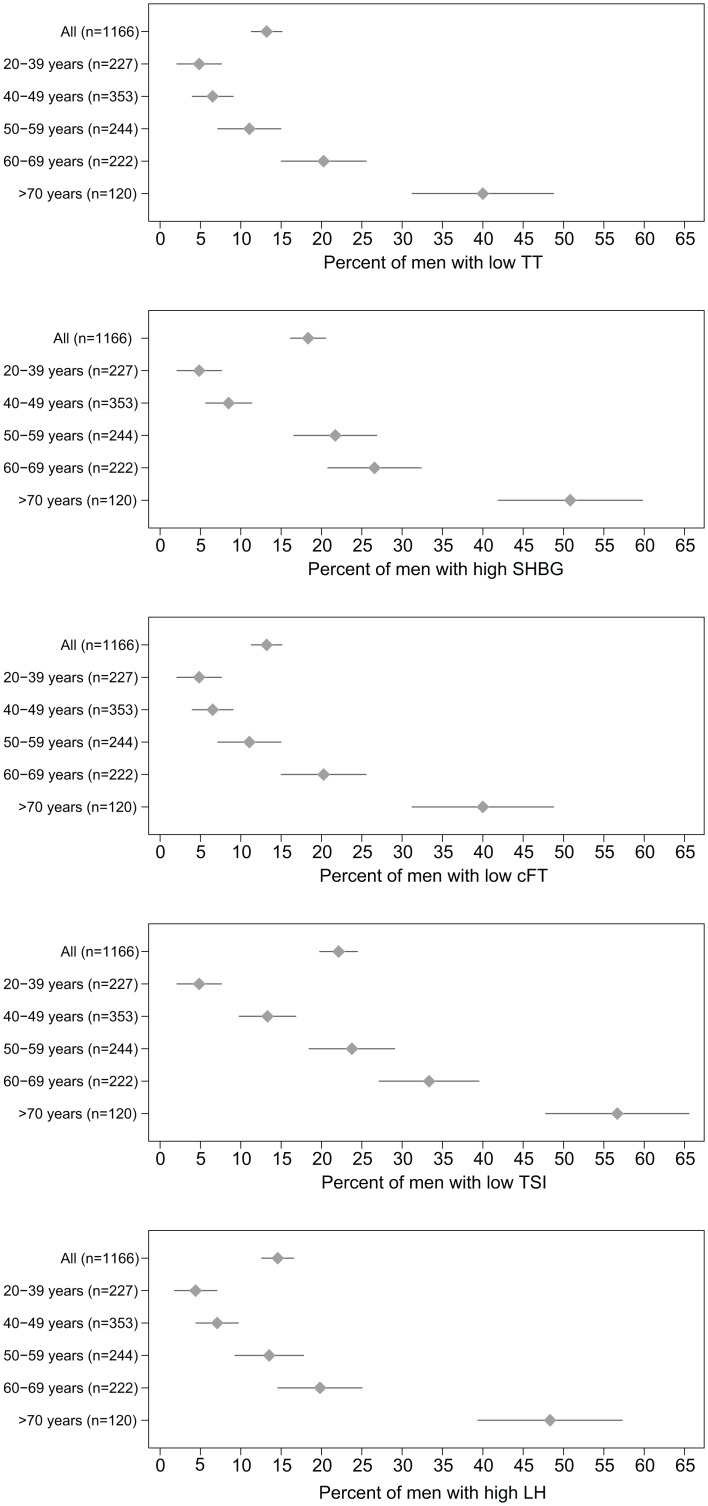
Percent abnormal hormone concentrations by age among older men. Mean (diamond) and 95% confidence interval (line) of the percent abnormal hormone concentrations among men, by decade of age, based on cutoff values shown in Table 2.

The percent of older adults below the reference range for TT, cFT and TSI was 3.8% (95% CI: 3.2%, 4.4%), 15.2% (95% CI: 14.1%, 16.3%), and 26.3% (95% CI: 24.9%, 27.8%), respectively. The percent of older adults higher than the reference range for SHBG and LH was 21.6% (95% CI: 20.2%, 23.0%) and 6.1% (95% CI: 5.3%, 6.9%) respectively. Graphs of the percent abnormal concentrations by age are shown in Fig 1. The percent of abnormal concentrations for TT does not change with age (p = 0.632, likelihood ratio test); however the percentage of abnormal SHBG (p<0.001), cFT (p<0.001), TSI (p<0.001), and LH (p<0.001) increase significantly with increasing age.

### Unadjusted Associations of Hormones with Lifestyle/Demographics

We compared hormone concentrations across categories of demographic and lifestyle variables (Table 3). TT was significantly associated with WHR, BG, SBP, farming, and marital status; notably, TT was not significantly associated with age. SHBG was significantly associated with age, WHR, farming, education, smoking, and alcohol use. cFT was associated with age, BG, SBP, farming and education. TSI was significantly associated with age, WHR, BG, SBP, farming, and education. There was a statistically significant association of LH with age, WHR, SBP, farming, education, marital status, and alcohol use.

**Fig 2:**
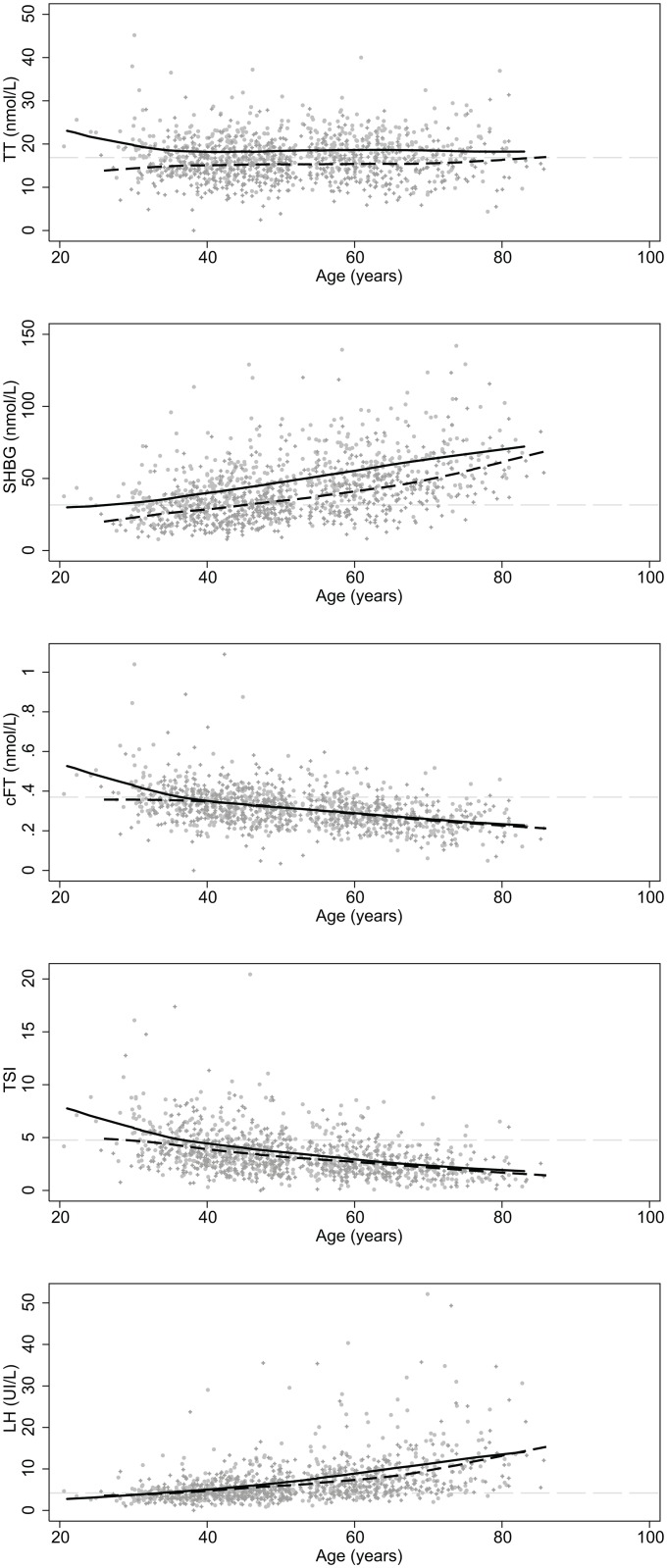
Change in hormone concentration with age, stratified by waist-to-height ratio. These are smoothed lowess curves where the solid line is WHR ≤ 0.5 and the dashed line is WHR > 0.5. Individual observations are represented with circle (WHR ≤ 0.5) and plus (WHR >0.5) markers. The reference line indicates the reference hormone concentration (the mean hormone concentration among 20–39 year olds). TT = total testosterone (reference = 16.88 nmol/L); SBGH = sex binding globulin hormone (reference = 31.67 nmol/L); cFT = calculated free testosterone (reference = 0.37 nmol/L); TSI = testosterone secreting index (reference = 4.76); LH = luteinizing hormone (reference = 4.20 IU/L).

Trends in hormone concentrations by age shown in Fig 2; these are stratified by WHR (≤0.5 versus >0.5). There is a statistically significant decrease in TT (*p≤*0.0001) and SHBG (*p*≤0.001) among men with a higher WHR; differences for other hormones may vary depending on age.

### Adjusted Associations of Hormones with Lifestyle/Demographics

Results from models predicting hormone concentrations by age, are presented in Table 4; these are adjusted for WHR category, blood glucose, SBP, farming vs. not, education level, marital status, smoking status, alcohol use, and history of vasectomy. In the adjusted model, there was a statistically significant decrease in TT related to higher WHR (adjusted β (βadj) = -2.87; 95% confidence interval (95% CI): -3.41, -2.32), BG (βadj = -0.37; 95% CI: -0.54, -0.20), and SBP (βadj = -0.02; 95% CI: -0.04, -0.01); however, there was no association with age. SHBG increased with aging (βadj = 0.76; 95% CI: 0.68, 0.85), but was reduced with increasing WHR (βadj = -11.59; 95% CI: -13.59, -9.61), BG (βadj = -0.98; 95% CI: -1.61, -0.35), SBP (βadj = -0.14; 95% CI: -0.19,-0.09), and among famers (βadj = -2.66; 95% CI: -5.23, -0.09). For cFT, the only variable which had a statistically significant association was age (βadj = -0.0033; 95% CI: -0.0037, -0.0028). TSI decreased with increased age (βadj = -0.06; 95% CI: -0.07, -0.05) and higher WHR (βadj = -0.42; 95% CI: -0.64, -0.20). LH increased with aging (βadj = 0.17; 95% CI: 0.14, 0.19) and among men with less education (βadj = -1.34; 95% CI: -2.31, -0.38), but decreased among married men (βadj = 1.30; 95% CI: 0.21, 2.39), among men with higher WHR (βadj = -0.78; 95% CI: -1.33, -0.23).

**Table 4:** Multivariable linear regression coefficient (95% confidence interval) for hormone concentrations related to selected characteristics, n = 1166.

Variable	TT, nmol/L	SHBG, nmol/L	cFT, nmol/L	TSI, no units	LH, IU/L
Age	0.02 (-0.01, 0.04)	0.76 (0.68, 0.85)*	-0.0033 (-0.0037, -0.0028)*	-0.06 (-0.07, -0.05)*	0.17 (0.14, 0.19)*
WHR	-2.87 (-3.41, -2.32)*	-11.59 (-13.59, -9.61)*	-0.0035 (-0.0141, 0.0072)	-0.42 (-0.64, -0.20)*	-0.78 (-1.33, -0.23)*
BG	-0.37 (-0.54, -0.20)*	-0.98 (-1.61, -0.35)*	-0.0030 (-0.0064, 0.0004)	-0.04 (-0.11, 0.03)	-0.09 (-0.27, 0.10)
SBP	-0.02 (-0.04, -0.01)*	-0.14 (-0.19, -0.09)*	0.00005 (-0.0002, 0.0003)	-0.001 (-0.007, 0.005)	-0.01 (-0.02, 0.01)
Farmer	-0.63 (-1.34, 0.08)	-2.66 (-5.23, -0.09)*	0.00002 (-0.01383, 0.01378)	-0.03 (-0.31, 0.26)	-0.27 (-0.98, 0.44)
Less education	-0.60 (-1.56, 0.37)	-2.92 (-6.41, 0.57)	-0.0158 (-0.0346, 0.0028)	0.26 (-0.12, 0.65)	-1.34 (-2.31, -0.38)*
Married	0.79 (-0.30, 1.88)	0.23 (-3.70, 4.17)	0.0089 (-0.0121, 0.0301)	0.06 (-0.38, 0.49)	1.30 (0.21, 2.39)*
Smoking	0.50 (-0.16, 1.15)	2.00 (-0.39, 4.38)	-0.0035 (-0.0163, 0.0093)	0.01 (-0.26, 0.27)	-0.30 (-0.96, 0.36)
Alcohol use	-0.02 (-0.58, 0.54)	0.17 (-1.85, 2.19)	0.00004 (-0.01081, 0.01088)	0.12 (-0.10, 0.35)	-0.20 (-0.76, 0.36)
Vasectomy	0.33 (-0.69, 1.35)	-0.46 (-4.16, 3.23)	0.0035 (-0.0163, 0.0234)	0.27 (-0.14, 0.68)	-0.29 (-1.31, 0.73)

TT = total testosterone; SHBG = sex hormone-binding globulin; cFT = calculated free testosterone; TSI = testosterone secreting index; LH = luteinizing hormone; WHR = waist-to-height ratio; BG = Blood glucose; SBP = systolic blood pressure. The model for each hormone includes all variables listed in the table. Age is in years; WHR has no units and >0.5 is compared to ≤0.5; glucose is blood glucose in nmol/L, SBP is in mmHg. Farmer is compared to not farmer, Less education is < 5 years compared to ≥ 5 years, married is compared to not married (single, divorced, widowed), smoking and alcohol use are current compared to never or former use, vasectomy is compared to no vasectomy.

*Likelihood ratio test, p < 0.05.

## Discussion

This study was designed to assess mean and reference concentrations of androgen hormones and determine the relationship of androgen hormone concentrations with age and other demographic and lifestyle characteristics among in adult Western Chinese males. A major strength of this work is that it is a large study, which increases its external validity and that we were able to control for numerous potential confounding variables. This is also the first study to be completed in Western Chinese men, and one of only a handful completed in China: as such, this study provides valuable information regarding geographic variability in age-related androgen concentrations.

There are a few limitations to this study. As a cross-sectional study, our results do not provide direct evidence for causal factors for testosterone concentrations. The original study design was a stratified, population-based sample of adult males from Zunyi; however, as key data regarding recruitment and enrollment were not recorded, we are unable to calculate a response rate or unequivocally verify that our study population accurately reflects all adult males from Zunyi. Nonetheless, we did succeed in enrolling large population of adult males from a representative sample of geographic across the Zunyi region; as many sociodemographic variables correlated with geographic region it is likely that we were still able to achieve a population sample that reflects the variation seen among males in Zunyi. Additionally, in contrast to several other well-regarded studies in this area [16], we did not exclude participants on the basis of prostate cancer or immune diseases. As such, our population likely represents a somewhat less healthy sample of adults, and our results should be interpreted accordingly.

Some additional limitations lie within assessment of testosterone and other hormones. Our results were based on a single testosterone measurement, which may not reflect a long-term average concentration. However, we would expect any variation to be randomly distributed in the population, and as we have a large sample size we would expect that our average estimates still provide a reliable representation of testosterone concentrations within this population. Additionally, as there was a four-hour period over which blood samples were collected, it is possible that diurnal variation may have affected our measured concentrations. More specifically, we may have included some values that were slightly less than the peak testosterone concentrations. Notably, the timing of blood collection was unrelated to the participant’s age. Thus, while we might expect mean hormone concentrations overall to be somewhat lower and their variance increased by the inclusion of measurements that are just outside of the peak daily values, we would expect this to be independent of age. It is also possible that the increased variance may have somewhat reduced our power to detect changes in hormone concentrations; however, given that we were successful in enrolling a large sample of men, and that our protocols are consistent with recommendations from several major professional societies [16], the likely influence of blood collection timing on our results is likely to be minimal.

Testosterone production is regulated by the hypothalamic-pituitary-testicular axis. The hypothalamus releases gonadotropin-releasing hormone, which in turn triggers the pituitary gland to produce LH and follicle stimulating hormone (FSH). LH travels to the testis, where it interacts with Leydig cells to produce testosterone [41]. High concentrations of free testosterone will inhibit LH production [42, 43], but in conditions with decreased free testosterone LH production would be increased. Some testosterone becomes tightly bound to SHBG whereas the remainder is considered to be free testosterone, and is either loosely bound to albumin or unbound to proteins [36]. Free testosterone can enter cells and interact directly which androgen receptors; thus it is considered the bioavailable fraction, or the fraction responsible for the biological activity of testosterone [44].

Our analysis identified a cutoff value for the lower limit of cFT as 0.22 nmol/L, and for TT as 9.16 nmol/L. These are both similar to previously reported cutoff estimates [10, 45].

Our results indicated increases in average serum level for both SHBG and LH, a decrease in average serum level for cFT, a measure of free testosterone, associated with increasing age. Additionally, we observed that with increasing age there was an increase in the proportion of adults with SHBG or LH concentrations higher than the reference range and cFT or TSI concentrations below the reference range. There was no change in either the average serum level or proportion of individuals under the reference range for TT with increasing age. The true mechanism for this is still unknown. One possible explanation is that LH production increased as a result of lower FT. However, it has been shown that with aging the number of LH receptors decreases: thus, increased LH may not have a substantial change on the amount of testosterone produced [38]. Meanwhile, the simultaneous increase in SHBG would result in much of the LH-triggered testosterone production to be bound rather than free testosterone. Thus, although increased testosterone production was signaled, the result may be to maintain total testosterone rather than free testosterone concentrations.

Our main finding that we did not observe a significant reduction in serum level and the proportion of men with low TT with aging is not consistent with several large population studies. The Massachusetts Male Aging Study, the Baltimore Longitudinal Study of Aging and an European Male Aging Study observed a decline of TT with aging [8, 11, 19]. Similar results were evident in an unadjusted baseline analysis of 3200 men from 8 different European countries within the European Male Aging Study; however, this association was no longer statistically significant after adjusting for confounders [46]. Our results are consistent with several cross-sectional studies completed among male Japanese office workers [22], healthy adult Chinese men [21], and most recently, among 1093 healthy men from Shanghai, China [14]. It is possible that geographic variability in genetics or environment may contribute to the differences in TT trends with age across these studies. Another observation from our work is the significant decline in serum level and an increase in the proportion of men with low cFT, an indicator of free testosterone, with increased age. This observation is consistent with the above-mentioned studies, regardless of their geographic location [19, 21, 22, 46]. Additionally, all of these studies consistently demonstrate that decreases in free testosterone are much greater in magnitude compared to decreases in total testosterone, regardless of whether a TT decrease was statistically significant.

It is possible that increased weight may also play a role in affecting hormone concentrations. Our adjusted regression models show a strong association between WHR with reduced TT and SHBG, but not with cFT. Prior cross-sectional studies have also identified significant inverse correlations of BMI, WHR, or both with TT [12, 14, 15, 21, 46] or SHBG [13–15, 21, 46], although not all studies have found significant associations [22]. These results are also supported by results from longitudinal studies which found increased obesity was associated with decreased TT and SHBG while decreased obesity was associated with increased TT and SHBG [11, 47]. Several studies suggest a potential mechanism for these observations: obesity-induced increases in estrogen are thought to play a major role in determining negative feedback at the pituitary level, and insulin resistance may also contribute to the low testosterone levels seen in obese men [48–50]. For example, increased free estradiol in older men has been associated with decreased SHBG and increased free testosterone, without a change in TT [51]. This may represent an alternative or additional mechanism to the increased LH production referenced above.

No association of obesity with free testosterone was found in Japanese and Chinese cohorts [12, 22], similar to our results; however, both Wu *et al*. and Svartberg *et al*. report inverse, cross-sectional associations of free testosterone with BMI [15, 46]. Interestingly, these initial observations suggest that there may also be an Asian/non-Asian difference in association of free testosterone with obesity, but, as relatively fewer studies have explored obesity and free testosterone as opposed to TT, this should be confirmed with additional studies.

The 2010 Endocrine Society guidelines recommend using both a measurement of low serum TT concentrations and the presence of symptoms or signs to diagnose Androgen Deficiency Syndromes in men [52]. Free or bioavailable testosterone assessment is only recommended among those whose total testosterone is borderline or when an abnormality in SHBG is suspected [52]. Although TT has been widely used as a diagnostic tool in many areas, particularly Western nations [3, 10, 48], there is still debate as to whether this is the best option [11, 13, 18, 19]. Free testosterone may also be a better predictor of clinical symptoms compared to TT: a recent study by Antonio *et al*. analyzed over 3000 community-dwelling men from the European Aging Male study and found that men with low free testosterone but normal TT experienced hypogonadal symptoms whereas men with normal free testosterone but low TT did not [28]. However, there is some conflicting evidence regarding the predictive ability of free testosterone. Ramasamy *et al*. performed a chart review of over 3000 men attending an outpatient men’s health clinic and did not find any association of hypogonadal symptoms with free testosterone among men with near-normal TT [53].

Despite the lack of a clear trend in the associations and predictive ability of total versus free testosterone, evidence from Asian populations does appear to be consistent in identifying that free testosterone, but not total testosterone, has a significant inverse association with age [4, 21, 22]; this is also consistent with the present study. For these reasons free testosterone, specifically cFT, has been recommended for use as a diagnostic tool in place of TT for Asian populations [4, 22]. In addition, our results indicate that the only covariate that was significantly associated with a decline in cFT was aging, suggesting cFT in our population is not influenced by demographic and lifestyle factors such as obesity, diabetes, smoke and alcohol use. This was not true for the other testosterone measures we evaluated, and is further evidence that cFT may be a better predictor of age-related androgen decline, as clinicians would not need to account for confounding from other demographic and lifestyle factors. Taken together, this evidence suggests cFT would be a better potential marker than TT for age-related testosterone decline among Western Chinese men.

## Conclusions

In this population-based study of Western Chinese men, we found an independent association of reduced cFT concentrations with aging while serum TT concentrations did not change with age. Additionally, LH and SHBG concentrations significantly increased with age, while FTI and TSI concentrations significantly decreased with age. Central obesity was significantly and inversely correlated to serum TT and SHBG. Our data suggest cFT might be a good potential marker for age-related androgen deficiency in this population.

## Supporting Information

S1 File Questionnaire (Chinese/English). The entire questionnaire used in this project, in Chinese; questions used in the present analysis are also translated into English.
(PDF)
