# Correction: Reference Ranges and Association of Age and Lifestyle Characteristics with Testosterone, Sex Hormone Binding Globulin, and Luteinizing Hormone among 1166 Western Chinese Men

**DOI:** 10.1371/journal.pone.0168029

**Published:** 2016-12-22

**Authors:** Xubo Shen, Ruifeng Wang, Na Yu, Yongjun Shi, Honggang Li, Chengliang Xiong, Yan Li, Ellen M. Wells, Yuanzhong Zhou

Fig 1 appears incorrectly in the published article. The first panel, “Percent of men with low TT,” and third panel, “Percent of men with low cFT,” contain the same data. Please see the corrected Fig 1 here.

**Fig 1:**
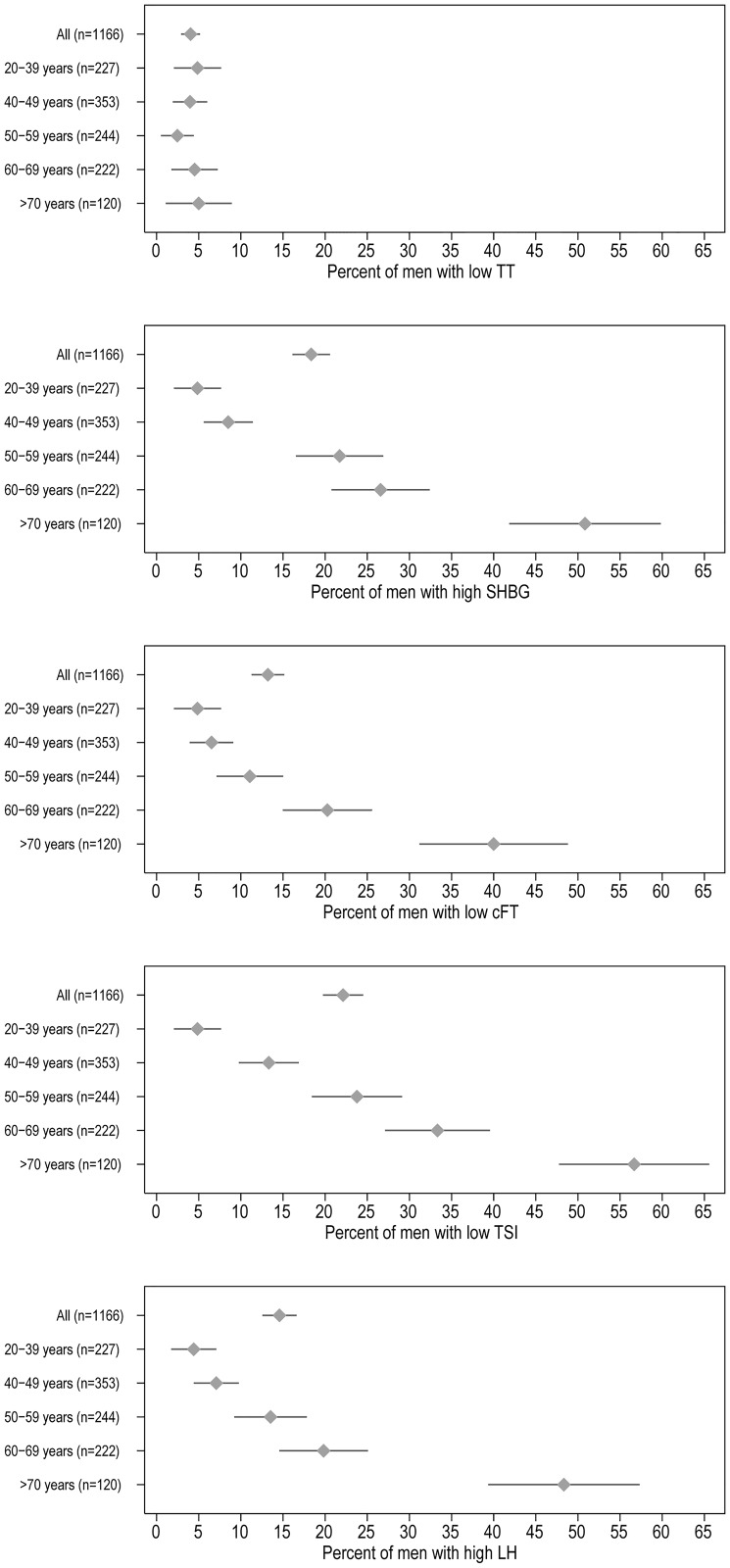
Percent abnormal hormone concentrations by age among older men. Mean (diamond) and 95% confidence interval (line) of the percent abnormal hormone concentrations among men, by decade of age, based on cutoff values shown in Table 2.
