# Supplementary material for: Reference Ranges and Association of Age and Lifestyle Characteristics with Testosterone, Sex Hormone Binding Globulin, and Luteinizing Hormone among 1166 Western Chinese Men
Source: PLoS One. 2016 Oct 6;11(10):e0164116. doi: 10.1371/journal.pone.0164116 (PMC5053410; doi:10.1371/journal.pone.0164116)
Supplement: S1 File — The entire questionnaire used in this project, in Chinese; questions used in the present analysis are also translated into English. (PDF) [file pone.0164116.s001.pdf]

调查对象编号：___________

知 情 同 意 书

您好！

此次体检的目的，是为了更好地了解中老年朋友生殖健康等方面的情况。调查内容包括健康查体和问卷两个部分。健康查体包括体格检查和抽血化验两部分，其中体格检查项目有身高体重、血压测量、心电图检测等，抽血化验项目为雄激素水平及相关基因检测；问卷用于评估您的一般健康状况及是否存在更年期症状，在您填写完成后由调查员统一填写回收。

全部调查成本合计约 500 元，我们对所有接受调查的中老年（40-79 岁）受访者免费，不收取任何费用；抽血检查的结果及健康状况评估也将及时反馈给您，使您对自己的健康状况有所了解。

我们对受访者个人信息严格保密，仅限于为健康检查出最终结论做参考，不会对您产生任何不良影响。请您为了健康体检结果的真实性如实填写或配合调查员填写。

如愿意接受问卷及健康检查，请在此处签名：

日期：　　　年　　　月　　　日

中国中老年男性生殖健康问卷

(Questionnaire for Chinese middle-aged male reproductive health)

（本页信息由医生填写）

Q0001	调查点代码<br>1. 北京 2. 江苏 3. 广东<br>4. 湖北 5. 陕西 6. 贵州	□
Q0002	地区类型代码(Type Code)<br>1. 城市(urban)<br>2. 农村(rural)	□
Q0003	调查对象编号:	□□□□□□
Q0004	调查员编号:	□
Q0005	调查员姓名:	______
Q0006	调查完成日期(年/月/日):	□□/□□/□□
Q0007	督导员审核日期(年/月/日):	□□/□□/□□
	督导员签字	______

“十二五”国家科技支撑计划项目

课题编码 2012BAI32B03

2013.1

第一部分　基本情况

(Demographic characteristic)

填写答案处

A1	您的年龄______（岁）(以身份证为准，至调查日止)<br>Age________(years)(according to your ID, to the date of investigation)	☐☐
A2	您的民族：　1-汉族　2-其他民族（请注明）____________<br>**nationality : l- Han　2- other ethnic (please note)____________**	☐
A3	您的文化程度是：<br>1-文盲　2-小学　3-中学或中专　4-大专及以上<br>**education :**<br>**1-Illiteracy 2-Primary(1-5years) 3-middle school to high school(6-11 years)　4- college and above(12 years and above)**	☐
A4	最近 5 年您主要居住在<br>1-地区及以上级别城市　2-县城　3-场镇或集镇　4-农村<br>**The primary residence regions in the last five years:**<br>**1-city higher than county –level 2- county -level city**<br>**3- town 4- rural**	☐
A5	您退休前的主要职业（或当前的职业）是：<br>1-公务员　2-工人　3-农民<br>4-教师　5-医护工作者　6-其他___________（请注明）<br>**Occupation:**<br>**1-Civil Servants 2-workers 3- farmers**<br>**4-teacher 5-Health care workers 6- others(please note)__________**	☐
A6	您当前的婚姻状况：<br>1-未婚　2-已婚（含同居）　3-离异　4-丧偶<br>**current marital status :**<br>**1- unmarried　2- married (including cohabitation)**<br>**3- divorced　4- widowed**	☐
A7	是否有亲生子女？<br>1-有　2-没有<br>**Have you offspring?**<br>**1-yes 2-no**	☐
A8	有没有接受过男性结扎手术？<br>1-有　2-没有<br>**Have you experienced vasectomy?**<br>**1-yes　2-no**	☐
A9	您的血型？　1-A 型　2-B 型　3-O 型　4-AB 型　5-不知道	☐
A10	参加哪种（哪些）类型医疗保险？（可多选）<br>1-城镇职工基本医疗保险　2-城镇居民基本医疗保险	☐

	3-新型农村合作医疗　　　　4-全公费<br>5-无　　　　　　　　　　　6-其他 ___________（**请注明**）	
A11	**是否吸烟**<br>1-从不吸烟（跳至 A14）　2-吸烟　3-戒烟（_______年）<br>**Smoking**<br>**1-never(skip to A14)　2-current smoking 3-quit from smoking**	☐
A12	**烟龄**<br>1-5 年及以内　2-6～10 年　3-11～15 年　4-16 年以上<br>**Years of smoking**<br>**1-5 years and below 2-6 years to 10 years**<br>**3-11 years to 15 years　4-16 years and above**	☐
A13	**吸烟量**<br>1-每天 10 支及以内　2-每天 11 支～20 支　3-每天 20 支以上<br>**Quantity of smoking**<br>**1-1 to 10 cigarettes 2- 11 to 20 cigarettes**<br>**3- more than 20 cigarettes**	☐
A14	**是否饮酒**<br>1-不饮酒（跳至 B1）　2-偶尔饮酒　3-经常饮酒　4-戒酒<br>**Alcohol use**<br>**1-never(skip to B1)　2- occasionally 3-often**<br>**4- quit from alcohol use**	☐
A15	**酒龄**<br>1-5 年及以内　2-6～10 年　3-11～15 年　4-16 年以上<br>**Years of alcohol use**<br>**1-5 years and below 2-6 years to 10 years**<br>**3-11 years to 15 years　4-16 years and above**	☐
A16	**饮酒量（近 1 年内）**<br>1-每周半斤以下　　2-每周半斤～2 斤　3-每周 2 斤以上<br>**（折合成 50 度白酒，注：啤酒 12 瓶可折合约 1 斤白酒）**<br>Quantity of drinking<br>1-Less than 250 gram alcohol per week<br>2-250 to 1000 gram alcohol per week<br>3- more than 1000 gram alcohol per week	☐

第二部分　家族史与既往史

(Family disease history and medical history)

填写答案处

B1	**疾病史：（可多选）**<br>1-无　2-糖尿病　3-心脏病　4-慢性支气管炎或哮喘	☐☐☐☐☐☐

	5-肿瘤　6-脑卒中　7-结核病　8-肝炎<br>9-前列腺癌　10-高血压　11-其他___________<br>Medical history:<br>1-no　2- diabetes 3- heart disease 4- chronic bronchitis or asthma 5- tumor 6- stroke 7- tuberculosis 8- hepatitis 9- prostate cancer 10- hypertension 11- Other
B2	泌尿生殖系统外伤、手术史：<br>1-无　2-有（限填 3 种）：________；________；________<br>Trauma and / or surgery of genitourinary system<br>1-no<br>2-yes(not more than 3 kinds):________；________；_____
B3	当前是否在使用某种长期使用的药物？<br>1-无　2-有，________________<br>Drug abuse<br>1-no 2-yes(please note)________
B4	父亲有无以下疾病史？（可多选）<br>1-无　2-糖尿病　3-心脏病　4-慢性支气管炎或哮喘<br>5-肿瘤　6-脑卒中　7-结核病　8-肝炎<br>9-前列腺癌　10-高血压　11-其他___________<br>Medical history of your father:<br>1-no　2- diabetes 3- heart disease 4- chronic bronchitis or asthma 5- tumor 6- stroke 7- tuberculosis 8- hepatitis 9- prostate cancer 10- hypertension 11- Other
B5	母亲有无以下疾病史？（可多选）<br>1-无　2-糖尿病　3-心脏病　4-慢性支气管炎或哮喘<br>5-肿瘤　6-脑卒中　7-结核病　8-肝炎<br>9-前列腺癌　10-高血压　11-其他___________<br>Medical history of your mother:<br>1-no　2- diabetes 3- heart disease 4- chronic bronchitis or asthma 5- tumor 6- stroke 7- tuberculosis 8- hepatitis 9- prostate cancer 10- hypertension 11- Other
B6	兄弟姐妹有无以下疾病史？（可多选）<br>1-无　2-糖尿病　3-心脏病　4-慢性支气管炎或哮喘<br>5-肿瘤　6-脑卒中　7-结核病　8-肝炎<br>9-前列腺癌　10-高血压　11-其他___________<br>Medical history of your sister and / or brother:<br>1-no　2- diabetes 3- heart disease 4- chronic bronchitis or asthma 5- tumor 6- stroke 7- tuberculosis 8- hepatitis 9- prostate cancer 10- hypertension 11- Other

第三部分　LOH 症状

（The Aging Males’ Symptoms (AMS) scale）

最近 3 个月内，您感觉：　　填写答案处

C1	感觉总体健康状况和精神状态下降（一般身体健康状况，主观感觉）<br>1一无症状　2一轻微　3一中度　　4一严重　5一非常严重	☐
C2	关节疼痛与肌肉疼痛（腰痛，关节痛，四肢痛，全背部痛）<br>1一无症状　2一轻微　3一中度　　4一严重　5一非常严重	☐
C3	多汗（无法预期的或是突然的流汗，並非劳累的情形下发生潮热<br>（突然感觉一阵发热，脸红或出汗））<br>1一无症状　2一轻微　3一中度　　4一严重　5一非常严重	☐
C4	睡眠障碍（入睡困难，易醒，早醒且感觉疲劳，睡眠质量差，失眠）<br>1一无症状　2一轻微　3一中度　　4一严重　5一非常严重	☐
C5	嗜睡，常常感觉疲乏无力<br>1一无症状　2一轻微　3一中度　　4一严重　5一非常严重	☐
C6	烦躁易怒：感觉容易冒犯和挑衅他人，容易为小事情感到心烦，情绪不稳定<br>1一无症状　2一轻微　3一中度　　4一严重　5一非常严重	
C7	神经质（如内心紧张，坐立不安，烦躁）<br>1一无症状　2一轻微　3一中度　　4一严重　5一非常严重	☐
C8	焦虑（如感觉到恐慌）<br>1一无症状　2一轻微　3一中度　　4一严重　5一非常严重	☐
C9	体力衰退/缺乏活力（整体表现下降，活动减少，对休闲活动缺乏兴趣，感到力不从心，成就感减少，必须勉强自己从事某些活动）<br>1一无症状　2一轻微　3一中度　　4一严重　5一非常严重	☐
C10	肌肉力量下降（感觉虚弱）<br>1一无症状　2一轻微　3一中度　　4一严重　5一非常严重	☐
C11	感觉压抑（情绪低落，悲伤，想哭（几乎要落泪），缺乏动力，情绪不稳定，觉得沒有任何事情是有意义的）<br>1一无症状　2一轻微　3一中度　　4一严重　5一非常严重	☐
C12	感觉已经过了人生的高峰期，开始走下坡路<br>1一无症状　2一轻微　3一中度　　4一严重　5一非常严重	☐
C13	感觉筋疲力尽，跌入人生谷底<br>1一无症状　2一轻微　3一中度　　4一严重　5一非常严重	☐
C14	胡须生长变慢或减少<br>1一无症状　2一轻微　3一中度　　4一严重　5一非常严重	☐
C15	性能力下降或性生活频率降低<br>1一无症状　2一轻微　3一中度　　4一严重　5一非常严重	☐
C16	早晨勃起的次数减少<br>1一无症状　2一轻微　3一中度　　4一严重　5一非常严重	☐
C17	性欲下降（性生活中的乐趣减少，对性生活的欲望减少）<br>1一无症状　2一轻微　3一中度　　4一严重　5一非常严重	☐
除上述症状外，您是否还有其他症状？如果有，请进行描述：		

第四部分　SF-36 量表

（The MOS 36-Item Short-Form Health Survey　）

填写答案处

D1	**总体来说，您认为自己的健康状况：**<br>1-非常好　2-很好　3-好　4-一般　5-差	☐
D2	**现在与一年前相比，您认为自己的总体健康状况：**<br>1-比一年前好很多　2-比一年前好一些<br>3-同一年前一样　4-比一年前差一些<br>5-比一年前差很多	☐
**您现在的身体状况影响您进行以下常规活动吗？如果影响，程度如何？**		
D3.1	剧烈的体力活动（如跑步、提举重物、参加高强度的体育运动）<br>1-受到很大影响　2-受到一些影响　3-完全不受影响	☐
D3.2	中等程度的体力活动（如移动桌椅，扫地，打太极拳，做体操等）<br>1-受到很大影响　2-受到一些影响　3-完全不受影响	☐
D3.3	上街购物时，拎起并携带日常用品等<br>1-受到很大影响　2-受到一些影响　3-完全不受影响	☐
D3.4	上三层或以上楼梯<br>1-受到很大影响　2-受到一些影响　3-完全不受影响	☐
D3.5	上一层楼梯<br>1-受到很大影响　2-受到一些影响　3-完全不受影响	☐
D3.6	弯腰或屈膝下蹲<br>1-受到很大影响　2-受到一些影响　3-完全不受影响	☐
D3.7	步行 1500 米以上<br>1-受到很大影响　2-受到一些影响　3-完全不受影响	☐
D3.8	步行 800 米<br>1-受到很大影响　2-受到一些影响　3-完全不受影响	☐
D3.9	步行 100 米<br>1-受到很大影响　2-受到一些影响　3-完全不受影响	☐
D3.10	自己洗澡或穿衣<br>1-受到很大影响　2-受到一些影响　3-完全不受影响	☐
**最近 4 周，您在工作和日常活动中是否因为身体健康原因而出现以下这些问题？**		
D4.1	减少了工作或其它活动的时间<br>1-是　2-否	☐
D4.2	本来想做的事情只能完成一部分<br>1-是　2-否	☐
D4.3	在工作或活动的种类上受到限制<br>1-是　2-否	☐
D4.4	在完成工作或其它活动时感到困难（比如需要更多的努力）<br>1-是　2-否	☐
**最近 4 周，您的工作或日常活动是否因情绪波动(消沉、忧虑等)出现以下问题？**		

D5.1	减少了工作或其它活动时间<br>1-是　　2-否	☐
D5.2	本来想做的事情只能完成一部分<br>1-是　　2-否	☐
D5.3	不能像往常那样认真完成工作或其他活动<br>1-是　　2-否	☐
D6	**最近 4 周,您的身体或情绪问题在多大程度上影响了您与家庭、朋友、邻居或集体中的社会交往?**<br>1-未受影响　　2-轻度影响　　3-中度影响<br>4-很大影响　　5-非常严重的影响	☐
D7	**最近 4 周，您有身体疼痛程度吗?**<br>1-没有疼痛　　2-极轻的疼痛　　3-轻度疼痛<br>4-中度疼痛　　5-重度疼痛　　6-非常剧烈的疼痛	☐
D8	**最近 4 周，疼痛对您的日常工作（包括工作及家务活动）的影响程度如何?**<br>1-未受影响　　2-轻度影响　　3-中度影响<br>4-很大影响　　5-非常严重的影响	☐
**请描述最近 4 周您有多少时间有下列感觉：**		
D9.1	感觉生活很充实<br>1-所有时间　　2-绝大部分时间　　3-大部分时间<br>4-有时　　5-很少时间　　6-从来没有	☐
D9.2	觉得是一个敏感的人<br>1-所有时间　　2-绝大部分时间　　3-大部分时间<br>4-有时　　5-很少时间　　6-从来没有	☐
D9.3	情绪非常低落，没有事情能使您高兴起来<br>1-所有时间　　2-绝大部分时间　　3-大部分时间<br>4-有时　　5-很少时间　　6-从来没有	☐
D9.4	感觉安静而平和<br>1-所有时间　　2-绝大部分时间　　3-大部分时间<br>4-有时　　5-很少时间　　6-从来没有	☐
D9.5	做事精力充沛<br>1-所有时间　　2-绝大部分时间　　3-大部分时间<br>4-有时　　5-很少时间　　6-从来没有	☐
D9.6	情绪低落<br>1-所有时间　　2-绝大部分时间　　3-大部分时间<br>4-有时　　5-很少时间　　6-从来没有	☐
D9.7	感觉精疲力竭<br>1-所有时间　　2-绝大部分时间　　3-大部分时间<br>4-有时　　5-很少时间　　6-从来没有	☐
D9.8	感觉快乐<br>1-所有时间　　2-绝大部分时间　　3-大部分时间<br>4-有时　　5-很少时间　　6-从来没有	☐
D9.9	感觉很厌烦<br>1-所有时间　　2-绝大部分时间　　3-大部分时间	☐

	4-有时　　　5-很少时间　　　6-从来没有
D10	**最近 4 周，有多少时间因为您的身体或情绪问题影响您的社会活动（如走亲访友等）？**<br>1-所有时间　　2-大部分时间　　3-有时候<br>4-很少时间　　5-从来没有　☐
**对下列情形选择最符合您情况的描述**	
D11.1	我似乎比别人更容易得病<br>1-完全符合　2-基本符合　3-不知道　4-基本不符合　5-从来没有　☐
D11.2	我像周围的人一样健康<br>1-完全符合　2-基本符合　3-不知道　4-基本不符合　5-从来没有　☐
D11.3	我觉得我的健康状况将变得更差<br>1-完全符合　2-基本符合　3-不知道　4-基本不符合　5-从来没有　☐
D11.4	我的健康状况非常好<br>1-完全符合　2-基本符合　3-不知道　4-基本不符合　5-从来没有　☐

第五部分　BECK 抑郁量表

（Beck depression inventory）

填写答案处

E1	0-我不感到忧郁　　1-我感到忧郁或沮丧<br>2-我整天忧郁，无法摆脱　　3-我十分忧郁，已经忍受不住　☐
E2	0-我对未来并不悲观失望　　1-我感到前途不太乐观<br>2-我感到我对前途不抱希望　　3-我感到今后毫无希望，不可能有所好转　☐
E3	0-我并无失败的感觉　　1-我觉得和大多数人相比我是失败的<br>2-回顾我的一生，我觉得那是一连串的失败<br>3-我觉得我是个彻底失败的人　☐
E4	0-我并不觉得有什么不满意　　1-我觉得我不能像平时那样享受生活<br>2-任何事情都不能使我感到满意一些　　3-我对所有的事情都不满意　☐
E5	0-我没有特殊的内疚感　　1-我有时感到内疚或觉得自己没价值<br>2-我感到非常内疚　　3-我觉得自己非常坏，一钱不值　☐
E6	0-我没有对自己感到失望　　1-我对自己感到失望<br>2-我讨厌自己　　3-我憎恨自己　☐
E7	0-我没有要伤害自己的想法　　1-我感到还是死掉的好<br>2-我考虑过自杀　　3-如果有机会，我还会杀了自己　☐
E8	0-我没失去和他人交往的兴趣<br>1-和平时相比，我和他人交往的兴趣有所减退<br>2-我已失去大部分和人交往的兴趣，我对他们没有感情<br>3-我对他人全无兴趣，也完全不理睬别人　☐
E9	0-我能像平时一样做出决断　　1-我尝试避免做决定<br>2-对我而言，做出决断十分困难　　3-我无法做出任何决断　☐
E10	0-我觉得我的形象一点也不比过去糟　☐

	1-我担心我看起来老了，不吸引人了<br>2-我觉得我的外表肯定变了，变得不具吸引力<br>3-我感到我的形象丑陋且讨人厌	
E11	0-我能像平时那样工作　1-我做事时，要花额外的努力才能开始<br>2-我必须努力强迫自己方能干事　3-我完全不能做事情	☐
E12	0-和以往相比，我并不容易疲倦　1-我比过去容易觉得疲乏<br>2-我做任何事都感到疲乏　3-我太易疲乏了，不能干任何事	☐
E13	0-我的胃口不比过去差　1-我的胃口没有过去那样好<br>2-现在我的胃口比过去差多了　3-我一点食欲都没有	☐

第六部分　实验室检查结果

（test of andorgen）

（以下由医生填写）

D1	血清总睾酮（TT）	
D2	SH性激素结合睾酮（SHBG）	
D3	推算的游离睾酮(CFT)	
D4	游离睾酮指数(FTI，TT/SHBG)	
D5	睾酮分泌指数（TSI,TT/LH）	
D6	黄体生成素（LH）	
D7		
D8		

贴化验单处：

健 康 体 检 表

健 康 体 检 表

Physical

姓名：________________　　　　　　出生日期：________年_______月

住址：____________________________________电话：____________________

工作单位：____

检查项目	检测值	工作人员签名
抽血（blood）		
问卷（questionnaire）		
身高（height）	________cm	
体重（weight）	________kg	
胸围（chest circle）	________cm	
腹围（abdomen circle）	________cm	
血压(blood pressure)	收缩压/舒张压____/____mmHg	
血糖（glucose）		
